# Compared Dexmedetomidine and Fentanyl on Sensory-Motor Block in Unilateral Intrathecal Anesthesia of Lower-limbs Orthopedic Surgeries: A Randomized Duble-blind Trials: Dexmedetomidine Versus Fentanyl on Sensory-motor Block in Unilateral Intrathecal Anesthesia

**DOI:** 10.31661/gmj.v11i.2499

**Published:** 2022-12-31

**Authors:** Mohammad Alipour, Seyed Javad Purafzali Firouzabadi, Atefeh Shahroudi, Mona Najaf Najafi, Ehsan Molaei

**Affiliations:** ^1^ Department of Anesthesiology, Ghaem Hospital, Mashhad University of Medical Sciences, Mashhad, Iran; ^2^ Student Research Committee, Department of Anesthesiology and Critical Care, Faculty of Medicine, Mashhad University of Medical Sciences, Mashhad, Iran; ^3^ Clinical Research Unit, Mashhad University of Medical Sciences, Mashhad, Iran

**Keywords:** Unilateral Intrathecal Anesthesia, Sensory-motor Block, Dexmedetomidine, Bupivacaine, Fentanyl

## Abstract

**Background:**

One of the alternatives for lower-limb orthopedic surgery is spinal anesthesia. It can affect the hemodynamic status and cause the prolonged motor and sensory blocks, as well as urinary retention, which are less common in the unilateral technique. Different drugs are used to improve the quality of the block and reduce its complications. Dexmedetomidine, a selective alpha-2 adrenergic receptor agonist, and fentanyl, an opioid medication, could administration as an adjuvant to increase the intrathecal block quality. Hence, this study aimed to compare unilateral spinal anesthesia with bupivacaine/dexmedetomidine (BD) and bupivacaine/fentanyl (BF) regimes on the sensory-motor block among patients with lower-limb orthopedic surgeries.

**Materials and Method:**

This randomized, double-blind clinical trial was performed on 36 patients who underwent lower-limb orthopedic surgeries in Qaem Hospital, Mashhad, Iran. The patients were randomly divided into two groups. Patients who received 5.7 mg hyperbaric bupivacaine 0.5% plus 10 μg fentanyl (BF group) or 5 μg dexmedetomidine (BD group) were administered for inducing unilateral spinal anesthesia. Patients and investigators responsible for data collection were not awarded from allocation groups. The sensory-motor block level, duration, postoperative analgesia, and complications were recorded and compared between the two groups.

**Results:**

No significant difference was observed between the two groups in hemodynamic changes (i.e., systolic and diastolic blood pressure and heart rate) before and after the blockage (P˃0.05). Also, there was no difference in the sensory-motor block level and anesthesia-related complications between BF and BD groups (P˃0.05).

**Conclusion:**

In patients for whom the use of opioids for unilateral spinal anesthesia is contraindicated, dexmedetomidine could be considered an appropriate alternative.

## Introduction

Spinal anesthesia is an appropriate choice for lower-limb orthopedic surgical procedures. However, it may affect the hemodynamic status and prolong the sensory-motor block and urinary retention, while those are less common in the unilateral technique [1]. Moreover, the patient generally has a more rapid return to the preoperative functional level, and recovery occurs faster [1]. In the unilateral method, as the patient is placed on the bed in the lateral decubitus position, a local hyperbaric anesthetic is injected into the subarachnoid space to limit the spread of the sensory-motor and sympathetic block [2]. Various factors, including the type, volume, and baricity of the medications used, as well as the type of needle and its bevel direction, can affect the success rate of unilateral spinal anesthesia [3].

Among the local anesthetics, bupivacaine is more commonly used for spinal anesthesia. The volume and dosage of bupivacaine used in unilateral spinal anesthesia vary from that of bilateral spinal anesthesia [4]. Clinicians used adjuvants such as opioids, alpha-2-adrenergic receptor agonist, magnesium sulfate, neostigmine, and midazolam to improve the block quality and increase its duration, besides reducing the dose of local anesthetics [5].

Recent studies indicated that dexmedetomidine-an alpha2-adrenergic receptor agonist-as an adjuvant could provide sufficient blockade during the operation, and the desired sedation post-operatively [6][7][8]. However, due to its high hemodynamic stability, it is more commonly used in intensive care units [9]. Although fentanyl (an opioid), as the adjuvant, can improve the intrathecal block quality, it can result in pruritus and respiratory depression [6].

This study aimed to compare unilateral spinal anesthesia's effect with bupivacaine/dexmedetomidine versus bupivacaine/fentanyl on the sensory-motor block level, duration, and postoperative analgesia. The hemodynamic changes in the patients were also taken under observation.

## Materials and Methods

### Patients and Study Design

This randomized, double-blinded clinical trial was conducted on patients referred to Qaem Hospital, a tertiary-level referral center affiliated with Mashhad University of Medical Sciences, Mashhad, Iran. All the patients underwent unilateral orthopedic surgery of the lower limb (e.g., knee or hip arthroplasty).

### Sample Size Calculation

The sample size was estimated according to Mahendru et al. study published in 2013 using Power Analysis and Sample Size (PASS) software version 11 for Windows (NCSS, LLC. Kaysville, Utah, USA) [7]. Considering α=0.05, power=90%, and β=0.1, a minimum of 14 patients for each group were calculated. By considering a possible dropout of 20%, a final patient number equal to 18 was indicated for each group.

### Inclusion and Exclusion Criteria

All patients with the American Society of Anesthesiologists (ASA) class I and II, aged over 18 years old, candied for unilateral orthopedic surgeries, and no need for general anesthesia were enrolled. Also, patients with increased intracranial pressure, infection at the blockade site, coagulopathy, cerebrospinal diseases, sensory system diseases, neuropathy, and a history of lumbar discopathy surgery were excluded from the study. Their expected surgical time was predicted as 90-120 min. Hence, all patients encountering perioperative adverse events such as excessive bleeding or prolonged surgical time were excluded.

### Ethical Issues

The study protocol was fully described to all patients, and written informed consent was obtained from each participant prior to the study. This study was approved by the Ethics committee of Mashhad University of Medical Sciences (code: IR.MUMS.REC.1395.285). Also, the study was registered in the Iranian Registry of Clinical Trials (registration code: IRCT20190510043545N1; available at: https://www.irct.ir/trial/39523).

### Study Protocol

The patines received bupivacaine/fentanyl (BF group), whereas bupivacaine/dexmedetomidine (BD group) was administered for unilateral intrathecal anesthesia. After monitoring and recording the baseline vital signs, performing a neurological examination, and obtaining a central venous line, 7 ml/kg of a crystalloid fluid (normal saline/ringer lactate) was administered, and the patient was placed in the lateral decubitus position on the side intended for surgery. A 25-gauge Quincke needle was then inserted into the L3-L4 intercostal space, and after entering the subarachnoid space, the needle's bevel was turned downwards. A 5.7 mg hyperbaric bupivacaine 0.5% and 10 μg fentanyl was injected in the BF group; accordingly, 5.7 mg hyperbaric bupivacaine 0.5% plus 5 μg dexmedetomidine was injected for the BD group. The patient remained in the mentioned position for 20 min before returning to the supine position. The changes in blood pressure and heart rate were instantly recorded, and the onset of motor and sensory blocks along with the sensory-motor level. In order to check the level of sensory block, a cold object was held in contact with the skin. The Bromage scale (Table-1) was used to check the accuracy of the motor block.

The patients were also studied for common complications, including pruritus, nausea and vomiting, and urinary retention. These factors were recorded every 5 min up to 30 min, and then every 15 min up to surgery termination. In the recovery unit, the same parameters were measured and recorded at the entrance as well as every 15 min up to the complete recovery of the motor and sensory block.

### Blinding and Randomization

Patients were randomized into BF or BD groups (n=18) according to a random number table. The trial was a double-blind design. All the spinal anesthesia was done by a single anesthesiologist who was aware of the prescribed medications, which was not involved in other sections of the study. Hence, patients and investigators responsible for data collection and analysis were unaware of group allocation and did not participate in the intervention during the trial.

### Statistical Analysis

The collected data were then analyzed using SPSS Statistics for Windows, version 16 (SPSS Inc., Chicago, Ill., USA). Fisher's exact test was used to compare the complications between the two groups. The mean sensory-motor block length and the time to block onset were compared by independent t-test and Mann-Whitney test. Repeated measures of ANOVA and Friedman tests were applied to study the hemodynamic changes in each group. The significance level was set at P<0.05.

## Results

**Figure-1:**
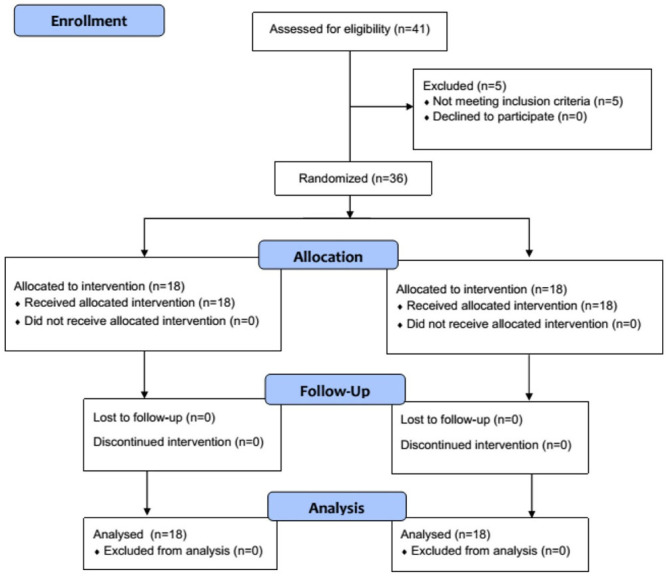
Figure 1. CONSORT flow diagram

**Figure-2:**
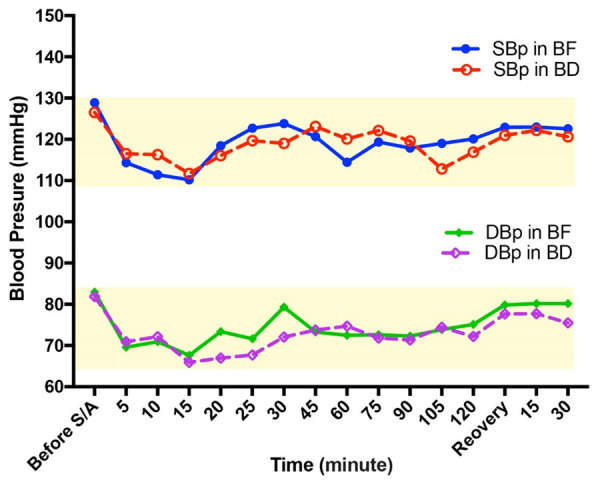
Figure 2. Mean Systolic and Diastolic Blood Pressure at Different Time Points in both Groups S/A: Spinal Anesthesia; SBp: Systolic Blood Pressure; DBp: Diastolic Blood Pressure; BF: Bupivacaine/fentanyl group; BD: Bupivacaine/dexmedetomidine group

**Figure-3:**
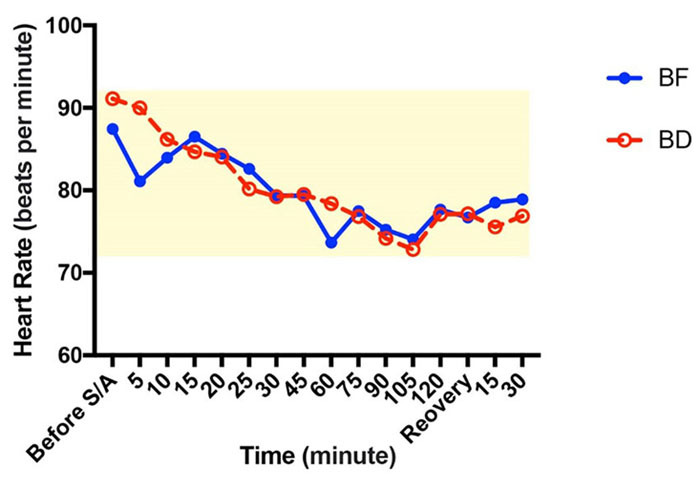
Figure 3. Mean Heart Rate at Different Time Points in both Groups S/A: Spinal Anesthesia; SBp: Systolic Blood Pressure; DBp: Diastolic Blood Pressure; BF: Bupivacaine/fentanyl group; BD: Bupivacaine/dexmedetomidine group

**Table:** Table1. Bromage Scale

**Grade**	**Criteria**	**Degree of block**
**I**	Free movement of legs and feet	Nil (0%)
**II**	Just able to flex knees with free movement of feet	Partial (33%)
**III**	Unable to flex knees, but with free movement of feet	Almost complete (66%)
**IV**	Unable to move legs or feet	Complete (100%)

**Table:** Table2. Baseline Charestestics of Studied Patients

**Variables**	**Groups**		**P-value**
	**BF (n=18)**	**BD (n=18)**	
**Sex, n(%)**			
Male	10 (55.5)	10 (55.5)	1
Female	8 (44.5)	8 (44.5)	
**Age, y (mean±SD)**	38.78±7.99	36.5±12.69	0.52
**BMI, Kg/m^2^ (mean±SD) **	25.46±2.33	23.88±3.86	0.14

**BF:** Bupivacaine/fentanyl; **BD:** Bupivacaine/dexmedetomidine; **BMI:** Body mass index

In total, 41 patients who were eligible enrolled and five patients due to not meeting inclusion criteria, were excluded from the study, and 36 patients were considered for final analysis (Figure-1). The mean age of patients in the BF and BD groups was 38.78±7.99 and 36.5±12.69 years. The baseline data of patients are presented in Table-2. Regarding Table-2, there were no any significant differences between the two groups in terms of sex, age, and body mass index (P˃0.05).

Before spinal anesthesia, the systolic blood pressure was 126.5±12.5 and 128.89±11.48 mmHg, and the diastolic blood pressure was 81.89±8.39 and 82.94±11.46 mmHg in the BD and BF groups, respectively, indicating no significant difference between the two groups (P˃0.05, Figure-2). The heart rate in the BD and BF groups was 87±8 and 91±9 bpm, respectively. These variables were recorded at 5, 10, 15, 20, 25, 30, 45, 60, 75, 90, 105, and 120 min after spinal anesthesia, at the recovery unit entrance, and 15 and 30 min after entering the recovery unit. All the data indicated no meaningful difference between the two groups in terms of hemodynamic stability (P˃0.05, Figure-3).

Motor block was studied based on the Bromage score at the same mentioned time points; it showed no significant difference between the two groups. The same result was obtained for the sensory block. Moreover, no significant difference was observed in the anesthesia-related complications between the two groups at the studied time points (Table-3).

Five patients in the BD group and seven in the BF group required analgesics following the sensory-motor block regression, indicating no significant difference between the two groups (P=0.48). Pain score was also studied after complete recovery of the sensory-motor block, again revealing no meaningful difference between the two groups (P=0.38).

## Discussion

**Table:** Table3. The Prevalence of Spinal Anesthesia-related Complications at Different Time Points in the Two Groups

**Complications**						**Time checkpoints (minutes) **					
		0-20	25	30	45-75	90	105-120	Entering recovery	15	30	After neuraxial block
**Pruritus**	BF	0	1	1	0	1	0	0	0	0	0
	BD	0	0	0	0	1	0	0	0	1	0
**Nausea**	BF	0	0	0	0	0	0	0	0	0	0
	BD	0	0	1	0	0	0	0	0	0	0
**Vomiting**	BF	0	0	0	0	0	0	0	0	0	0
	BD	0	0	0	0	0	0	0	0	0	0
**Urinary reten-tion**	BF	0	0	1	0	0	0	0	0	0	0
	BD	0	0		0	0	0	0	0	0	0

**BF:** Bupivacaine/fentanyl; **BD:** Bupivacaine/dexmedetomidine

Spinal anesthesia is widely used for surgeries of the lower abdomen and the lower limbs. It has side effects such as hemodynamic status instability and delayed sensory-motor and urinary retention regression [1].

To date, there have been few studies on the application of dexmedetomidine as an adjuvant to bupivacaine in the unilateral spinal anesthesia technique. Fentanyl is a lipophilic opioid μ receptor agonist and induces its effect by binding to the opioid receptors of the posterior spinal horn [10]. On the other hand, dexmedetomidine is a selective alpha-2-adrenergic agonist that binds to the presynaptic c-fiber and the post-synaptic posterior horn [10]. Herein, we investigated fentanyl and dexmedetomidine in unilateral spinal anesthesia and compared them with similar studies in the available literature.

In the present study, heart rate, systolic and diastolic blood pressures were measured before and after spinal anesthesia and peri- and post-operatively, in which no significant difference was observed between the two groups. In the study by Nayagam et al., similar results were obtained for standard spinal anesthesia [11]. Mahendru et al. also compared mean arterial blood pressure between the two groups with the same technique and reported no meaningful difference [7]. However, in

Khan et al. study, systolic blood pressure and heart rate were significantly lower in the BD group by the standard spinal anesthesia technique [6]. In contrast, the diastolic blood pressure showed no significant difference.

In the current study, sensory block regression revealed no significant difference between the two groups. Khan et al. [6] and Gupta et al. studies [10] confirmed our findings for standard spinal anesthesia. However, sensory-motor block in Mahendru et al. study [7] and Basuni et al. study [12] showed a delayed recovery in the BD group with the standard spinal anesthesia technique. In our study, motor block regression was not significantly different between the two groups, similar to sensory block. Regarding spinal anesthesia side effects, no difference was observed between the two groups. Other studies have confirmed our standard technique findings [6][7].

Regarding the need for analgesics following surgery and the pain score, no difference was observed in the present study between the two groups. In contrast, in Gupta et al. [1] study, the need for analgesics in the BD group was significantly less. Basuni et al. reported a significantly lower pain score in the BD group post-operatively [12].

Taher-Baneh et al. [13] compared the addition of fentanyl and dexmedetomidine to bupivacaine for unilateral spinal anesthesia. No significant difference was seen in the duration of sensory and motor block and unilateral complications between the BF and BD groups [13], which is similar to our findings.

Nevertheless, we cannot present a precise pattern for the exact time of sensory and motor block length in each group, which could be regarded as one of the main limitations of this study. The reason for this defect was that the surgery was ongoing, and we could not assess the level of sensory and motor block at any desired time. They could only be studied at the defined time intervals; unfortunately, we could not achieve a precise pattern for each group in this respect.

## Conclusion

The addition of dexmedetomidine to local anesthetics such as fentanyl in unilateral spinal anesthesia can result in the improved quality of the sensory and motor blocks and the reduced required dosage of local anesthetics. Therefore, in all patients in whom the application of opioids for unilateral spinal anesthesia is contraindicated due to any etiology, dexmedetomidine can be considered an appropriate alternative.

## Conflict of Interest

The authors declare no conflicts of interest.
